# Myrothecols G and H, Two New Analogues of the Marine-Derived Quinone Sesquiterpene Penicilliumin A

**DOI:** 10.3390/md13063360

**Published:** 2015-05-27

**Authors:** Ying Fu, Ping Wu, Jinghua Xue, Hanxiang Li, Xiaoyi Wei

**Affiliations:** 1 Key Laboratory of Plant Resources Conservation and Sustainable Utilization, South China Botanical Garden, Chinese Academy of Sciences, Xingke Road 723, Tianhe District, Guangzhou 510650, China; E-Mails: fuying@scib.ac.cn (Y.F.); wuping@scbg.ac.cn (P.W.); xuejh@scbg.ac.cn (J.X.); wxy@scbg.ac.cn (X.W.); 2 Guangdong Provincial Key Laboratory of Applied Botany, South China Botanical Garden, Chinese Academy of Sciences, Xingke Road 723, Tianhe District, Guangzhou 510650, China; 3 University of Chinese Academy of Sciences, Yuquanlu 19A, Beijing 100049, China

**Keywords:** 13-hydroxyl penicilliumin A, myrothecol G, myrothecol H, quinone sesquiterpenes, stereochemistry, theoretical conformational analysis, cytotoxicity

## Abstract

Two new quinone sesquiterpenes named myrothecols G and H (**1** and **2**), a pair of C-1′ diastereomers of 13-hydroxyl penicilliumin A, were isolated from the mycelia solid cultures of *Myrothecium* sp. SC0265. Their structures, including the absolute configurations, were established on the basis of the spectroscopic data combining with the theoretical conformational analysis. The cytotoxic activities of **1** and **2** were tested against a panel of human tumor cell lines.

## 1. Introduction

In recent years, secondary metabolites obtained from marine-derived fungi have gained considerable attention, as many of them are structurally unique and possess interesting biological properties [1]. Their structures have been successfully elucidated mainly by modern NMR techniques, and determination of the stereochemical relationships within every new molecule is generally one of the crucial points in structural determination. The configuration elucidation of natural products is of premiere importance because it provides essential information for both total synthesis and molecular mode of actions [2]. For example, quinine, originally isolated from the bark of cinchona trees, has been used for the treatment of malaria for centuries [3]. Quinidine, a stereoisomer of quinine, on the other hand, is used as a class Ι antiarrthythmic agent by prolonging the cardiac action potential [4].

**Figure 1:**
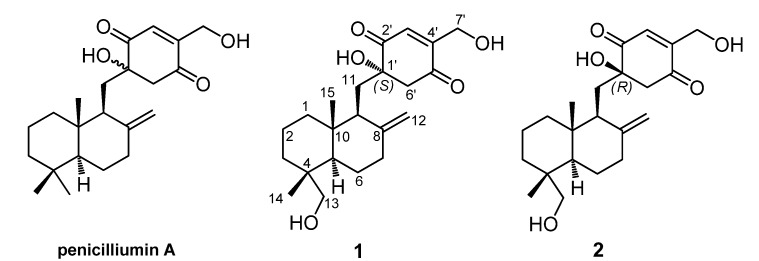
Penicilliumin A and two analogues (**1** and **2**) from *Myrothecium* sp. SC0265.

Penicilliumin A is a novel quinone-linked drimane sesquiterpene [5,6] produced by *Penicillium* sp. F00120 isolated from deep sea sediment sample [7], the absolute configuration of which has not been assigned yet. Our recent investigation on the secondary metabolites of *Myrothecium* sp. SC0265 led to the isolation of two new meroterpenoids, named myrothecols G (**1**) and H (**2**), which are structurally related to penicilliumin A (Figure 1). Herein, we presented the stereochemical assignment of the two new compounds by the detailed analysis of their spectroscopic data, especially 2D NMR spectra, combining with the theoretical conformational analysis.

## 2. Results and Discussion

Our previous investigation on the secondary metabolites of *Myrothecium* sp. SC0265 led to the isolation of a series of myrothecols, displayed potent cytotoxic and antibacterial activities [8]. This motivated us to search for minor components, and reinvestigation of this fungus yielded two new penicilliumin A analogues (**1** and **2**) via an NMR-guided fractionation process.

**Figure 2:**
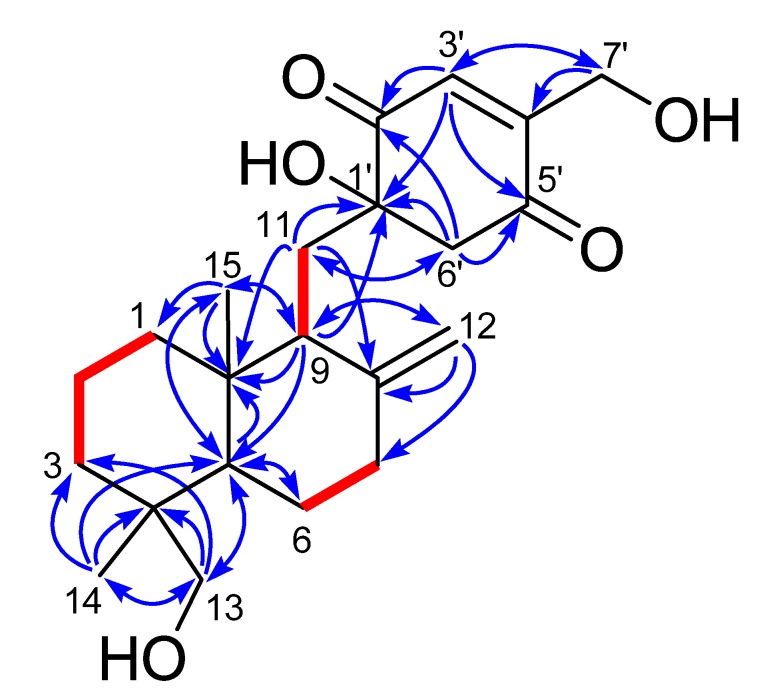
^1^H–^1^H COSY (bold lines) and key HMBC correlations (arrows) of **1** and **2**.

Compound **1** was obtained as yellow viscous oil and its HRESIMS spectrum showed a peak at *m*/*z* 399.2145 [M + Na]^+^ (calcd for C_22_H_32_NaO_5_, 399.2142) corresponding to the molecular formula C_22_H_32_O_5_ (seven unsaturations). ^1^H and ^13^C NMR spectra in combination with the HSQC analysis (Table 1) revealed the presence of two methyl groups, nine sp^3^ methylenes (two were oxygen-bearing), two sp^3^ methines, three sp^3^ quaternary carbons (one was oxygen-bearing), four olefinic carbons (one was terminally double-protonated) and two carbonyl carbons. In the ^1^H–^1^H COSY spectrum (Figure 2), three spin systems were confirmed: from C-1 to C-3; from C-5 to C-7; and from C-9 to C-11, as shown in Figure 2. The remaining degrees of unsaturation except two carbonyl groups and two olefinic bonds revealed the presence of three rings in 1. Only twenty-nine hydrogens directly connected to the carbon atoms in the HSQC spectrum indicated the existence of three hydroxyl groups, corresponding to the three oxygenated groups. HMBC correlations (Figure 2) from H_3_-14 to C-3, C-4, C-5 and C-13, and from H_2_-13 to C-3, C-4, C-5 and C-14, and from H-5 to C-10 and C-15, and H_3_-15 to C-1, C-5 and C-10 established a cyclohexane ring with a methyl group (Me-14) and an oxygenated methylene (δ_C_ 71.6, CH_2_OH-13) substituted at C-4, and a methyl group (Me-15) at C-10. Combining with the HMBC correlations from H-5 to C-6, and from H-9 to C-5, C-10 and C-15, and from H_2_-11 to C-8 and C-10, and H_2_-12 to C-7, C-8 and C-9, a drimane sesquiterpene skeleton was identified. A *p*-quinone moiety with a hydroxyl group at C-1ʹ (δ_C_ 78.1) and an oxygenated methylene (δ_C_ 58.7, CH_2_OH-7ʹ) at C-4ʹ, was established based on the HMBC correlations from H-3ʹ to C-1ʹ, C-5ʹ and C-7ʹ, and from H_2_-6ʹ to C-1ʹ, C-2ʹ and C-5ʹ, and H_2_-7ʹ to C-3ʹ and C-4ʹ. The *p*-quinone moiety was attached to the sesquiterpene ring at C-11 on account of the HMBC correlations from H-9 to C-1ʹ, and from H_2_-11 to C-1ʹ and C-6ʹ. These spectroscopic features indicated that **1** was a *p*-quinone-linked drimane sesquiterpene (Figure 1), similar to penicilliumin A, except for an additional hydroxyl group at C-13 in **1**.

The other co-isolated quinone sesquiterpene, named myrothecol H (**2**) had the same molecular formula (C_22_H_32_O_5_) as **1**, which was determined from HRESIMS data. Analysis of 1D NMR (Table 1), HSQC, ^1^H–^1^H COSY and HMBC spectra (Figure 2) of **2** revealed that **2** and **1** had the same planar structure. The main difference between **1** and **2** was the inconsistent ^13^C NMR data of C-11 (δ_C_ 32.5 in **1**; 35.5 in **2**) and C-6′ (δ_C_ 51.8 in **1**; 55.0 in **2**), suggested that **2** and **1** were a pair of epimers differing at the stereochemistry of C-1′.

The assignment of the relative configurations of the drimane sesquiterpene units in **1** and **2** were straightforward because of the observation of NOE correlations (NOEs) of H-5/H-9, H-5/H-13a (α-orientation) and H_3_-15/H-11a (β-orientation) in both of the NOESY spectra of **1** and **2**. The absolute configurations of the units in both compounds could be assigned to be the same as those of previously obtained myrothecols from the same strain by comparison with their NMR data and the biosynthesis consideration [8]. However, the sesquiterpene unit and the *p*-quinone moiety in **1** and **2** were connected by two single bonds (C-9–C-11 and C-11–C-1′), and the two compounds should exist as an equilibrium of multiple rotamers of the two bonds. Thus, the assignment of the configurational relationship between the sesquiterpene unit and the *p*-quinone moiety become a critical matter for the stereochemical assignment of the two compounds.

The NOEs of H-11a/H_3_-15, H-11b/H-1β, H-12a/H-7β and H-11a/H-12b observed in both of the NOESY spectra of **1** and **2**, and the *J*_9,11a_ value (7.3 Hz in **1**, 8.6 Hz in **2**) indicated that H-9 was nearly in an *anti* relationship with H-11a and in a *gauche* relationship with H-11b, thus C-9–C-11 bond was relatively fixed, and the flexibilities of **1** and **2** were mainly due to the rotation of C-11–C-1′ bond.

**Table 1:** NMR data (600/150 MHz, pyridine-*d*_5_) of compounds **1** and **2**.

	1		2	
Position	δ_C_, type	δ_H_, mult. (*J* in Hz)	δ_C_, type	δ_H_, mult. (*J* in Hz)
1α	38.9, CH_2_	1.31–1.36 m	38.9, CH_2_	1.47 dt (13.0, 3.0)
1β		1.76 br d (12.9)		1.76–1.83 overlapped
2α	19.5, CH_2_	1.40–1.44 m	19.6, CH_2_	1.52–1.57 m
2β		1.57 qt (13.4, 3.0)		1.61 qt (13.3. 3.0)
3α	36.3, CH_2_	1.83 m	36.5, CH_2_	1.79 overlapped
3β		1.38 br d (12.3)		1.40 dt (12.8, 3.1)
4	39.3, C		38.9, C	
5	49.2, CH	1.91 dd (12.9, 2.6)	49.2, CH_2_	1.76–1.83 overlapped
6α	24.9, CH_2_	1.81–1.86 overlapped	25.1, CH_2_	1.76–1.83 overlapped
6β		1.27–1.31 overlapped		1.26 qd (12.3, 4.1)
7α	38.8, CH_2_	2.04 td (12.9, 4.5)	38.8, CH_2_	2.02 td (12.5 5.0)
7β		2.34 dt (12.6, 3.0)		2.23–2.28 overlapped
8	150.5, C		149.7, C	
9	51.5, CH	2.43 br d (7.0)	51.2, CH	2.48 br d (8.5)
10	40.9, C		40.6, C	
11a	32.5, CH_2_	2.25 dd (15.1, 7.3)	35.5, CH_2_	2.27 dd (14.7, 8.7)
11b		2.24 br d (15.1)		2.21 br d (14.7)
12a	108.3, CH_2_	4.94 br s	107.5, CH_2_	4.81 br s
12b		4.71 br s		4.64 br s
13a	71.6, CH_2_	3.62 d (10.7)	71.7, CH_2_	3.54 d (10.6)
13b		3.29 d (10.7)		3.27 d (10.6)
14	18.4, CH_3_	0.79 s	18.2, CH_3_	0.78 s
15	15.9, CH_3_	0.72 s	16.0, CH_3_	0.69 s
1ʹ	78.1, C		78.8, C	
2ʹ	202.2, C		201.8, C	
3ʹ	134.5, CH	7.49 t (2.0)	135.4, CH	7.49 t (2.0)
4ʹ	154.9, C		153.7, C	
5ʹ	197.7, C		197.9, C	
6ʹα	51.8, CH_2_	3.24 d (16.3)	55.0, CH_2_	3.41 d (15.7)
6ʹβ		3.32 d (16.3)		3.47 d (15.7)
7ʹa	58.7, CH_2_	4.94 dd (18.6, 2.1)	58.8, CH_2_	5.01 dd (18.4, 2.1)
7′b		4.86 dd (18.6, 2.1)		4.85 dd (18.4, 2.1)

**Figure 3:**
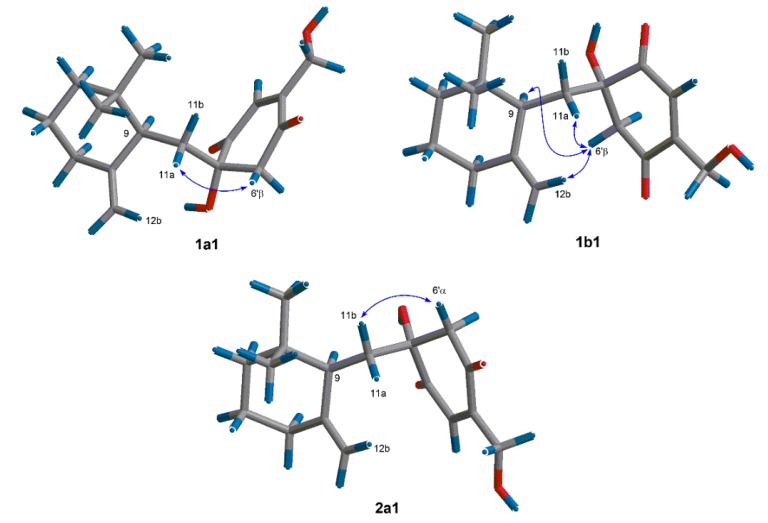
Dominant low-energy conformers (**1a1**, **1b1** and **2a1**) and key NOE correlations (curves) of **1** and **2**.

In order to find out the dominant low-energy conformers of **1** and **2**, 1′*S*- and 1′*R*-isomers were built and subjected to theoretical conformational analysis as previously described [8]. As a result (Table S1**)**, 1′*S*-isomer afforded three groups of low-energy conformers (**1a**, **1b** and **1c**), of which the group **1a**, represented by the global energy minimum **1a1** (Figure 3) accounted for 52.6% equilibrium population of the compound in MeOH solution according to Boltzmann statistics and the group **1b**, as represented by the second lowest energy minimum **1b1**, accounted for 44.8% equilibrium population, whereas, 1′*R*-isomer gave two groups of low-energy conformers (**2a** and **2b**) with the group **2a** (accounting for 86.8% equilibrium population) being dominant minima. The NOE correlation of H-6ʹβ/H-11a observed in the NOESY spectrum of **1** was corresponding with both of the conformers **1a1** and **1b1**, whereas the NOEs of H-6ʹβ/H-9 and H-6ʹβ/H-12b were distinctive of the conformer **1b1** (Figure 3). The situation in the NOESY spectrum of compound **2**, was that the NOE correlation of H-6ʹα/H-11b was clearly existed while the NOEs of H-6ʹα with H-12a and H-11a were absent, which was characteristic of the energy minima **2a** as shown for conformer **2a1** in Figure 3. Thus, **1** and **2** were assigned to have 1′*S* and 1′*R* configurations, respectively.

Considering that the main difference between **1** and **2** was the ^13^C NMR chemical shifts of C-11 and C-6′, the consistent ^13^C NMR data of C-11 (δ_C_ 34.7 in penicilliumin A; 35.5 in **2**) and C-6′ (δ_C_ 53.0 in penicilliumin A; 55.0 in **2**) suggested that the absolute configuration of penicilliumin A was the same as that of myrothecol H, although the NMR data of myrothecols G and H, and penicilliumin A were recorded in different deuterated solvents.

Since penicilliumin A showed cytotoxic activity against tumor cells [7], compounds **1** and **2** were evaluated for *in vitro* cytotoxicity against human lung adenocarcinoma A549, human hepatoma HepG2, and human cervical carcinoma HeLa cell lines by MTT method. Compounds **1** and **2** displayed weak cytotoxicity against the tested cell lines above. The IC_50_ values were presented in Table 2.

**Table 2:** Cytotoxicity (IC_50_, μM) *^a^* of compounds **1** and **2**.

	Cell Lines		
Compound	A549	HeLa	HepG2
**1**	46.7 ± 0.8	15.9 ± 0.8	31.9 ± 0.9
**2**	40.2 ± 1.5	28.7 ± 0.8	25.7 ± 1.6
Adriamycin *^b^*	0.69 ± 0.06	0.47 ± 0.05	1.22 ± 0.02

*^a^* Values represent means ± SD based on three individual experiments; *^b^* Positive control.

## 3. Experimental Section

### 3.1. General

Optical rotations were measured on a Perkin EImer 343 spectropolarimeter. UV measurements were conducted with a Perkin EImer Lambda 650 UV/VIS spectrometer. ^1^H NMR (600 MHz), ^13^C NMR (150 MHz), and 2D NMR spectra were recorded on a Bruker AV-600 instrument in pyridine-*d*_5_ with TMS as an internal reference. HRESIMS data were obtained on a Bruker Bio TOF IIIQ mass spectrometer in positive-ion mode. Preparative HPLC was performed by an HPLC system equipped with a Shimadzu LC-6AD pump and a Shimadzu RID-10A refractive index detector (Shimadzu Corporation, Kyoto, Japan) using a YMC-pack ODS-A C_18_ column (5 µm, 250 × 20 mm). For column chromatography, silica gel 60 (100–200 mesh, Qingdao Marine Chemical Ltd., Qingdao, China), Chromatoxex^℘^ ODS (C_18_, MB100 40~75 µm) were used. Thiazolyl Blue Tetrazolium Bromide (MTT) regent for cytotoxicity was purchased from Aladdin^℘^, Shanghai Jingchun Biochemical & Technologies Inc., shanghai, China. The cytotoxicity assay was determined with a Genois microplate reader (Tecan Group, Männedorf, Zürich, Switzerland).

### 3.2. Fungal Material and Fermentation

The producing fungus, *Myrothecium* sp. SC0265, was isolated from a forest leaf litter sample collected at the district of rare and endangered tree species in the Dinghu Mountain Biosphere Reserve, Guangdong, China, in March 2003. It was authenticated by its morphological characteristics and ITS DNA sequence data (Genbank accession number KM086710) by Prof. Tai-hui Li, Guangdong Institute of Microbiology, Guangzhou, China. Fermentation of the fungus was performed as previously described [8].

### 3.3. Extraction and Isolation

The mycelia culture was extracted with 95% EtOH, and the resultant extract was sequentially partitioned with petroleum ether, EtOAc, and *n*-BuOH. The EtOAc-soluble extract (28 g) was separated by silica gel CC and eluted with CHCl_3_–MeOH mixtures of increasing polarity (98:2–70:30) to afford eight primary fractions (F1–F8). Fraction F3, obtained on elution with CHCl_3_–MeOH (95:5), was then subjected to ODS CC using decreasing polar aqueous MeOH (40%–90%) to obtain seven fractions (F3-a–F3-g). Fraction F3-d, obtained from elution with 60% MeOH, was further separated by preparative HPLC (5 mL/min) using 60% MeOH to obtain **1** (29.0 mg, *t*_R_ = 17.9 min) and **2** (71.2 mg, *t*_R_ = 19.5 min).

**Compound 1**: yellow viscous oil; ${\left[\text{α}\right]}_{\text{D}}^{20}$ +15 (*c* 0.20, MeOH); UV (MeOH) λ_max_ (log ε) 202 (4.0), 238 (3.7); ^1^H and ^13^C NMR data, see Table 1; HRESIMS *m*/*z* 399.2145 [M + Na]^+^ (calcd for C_22_H_32_NaO_5_, 399.2142).

**Compound 2**: yellow viscous oil; ${\left[\text{α}\right]}_{\text{D}}^{20}$ +44 (*c* 0.93, MeOH); UV (MeOH) λ_max_ (log ε) 210 (4.0), 238 (3.8); ^1^H and ^13^C NMR data, see Table 1; HRESIMS *m*/*z* 399.2144 [M + Na]^+^ (calcd for C_22_H_32_NaO_5_, 399.2142).

### 3.4. Theoretical Conformational Analysis Method

Molecular Merck force field (MMFF) calculations and DFT calculations were performed with Spartan’14 software package (Wavefunction Inc., Irvine, CA, USA) [9], using default grids and convergence criteria. MMFF conformational searches were performed using Monte Carlo search method. The low-energy conformers within a 10 kcal/mol energy window were optimized using DFT method at the B3LYP/6-31G (d,p) level. Frequency calculations were run at the same level to estimate free energies at 298.15 K. Solvent effects were taken into account by using polarizable continuum model (PCM). In a preliminary set of calculations with compounds **1** and **2**, the truncated structure (the fragment from C-2 to C-4, including the methyl and hydroxymethyl groups attached at C-4, was removed from the intact structure and an H atom was added to C-1 and C-5) provided similar relative energies with the intact structure, and the SVP basis set gave consistent results with the TZVP one.

### 3.4. Cytotoxicity Assay

Human lung adenocarcinoma A549, human hepatoma HepG2, and human cervical carcinoma HeLa cell lines were obtained from Kunming Institute of Zoology, Chinese Academy of Sciences (Kunming, China). The cytotoxicity assay was conducted by MTT method [10].

## 4. Conclusions

In the case of quinone-drimane sesquiterpenes such as penicilliumin A, and myrothecols G and H (**1** and **2**), the *p*-quinone moiety and the drimane sesquiterpene unit were linked by two single bonds, which made the stereochemistry elucidation difficult because of the flexibility. The MMFF conformational search followed by geometry optimization using DFT method at B3LYP/6-31G (d,p) level on **1** and **2**, indicated that they existed the dominant low-energy conformers, which were flexible in a certain range and revealed their tracks by NOE correlations and the specific coupling constants. Thus, the stereochemistry of myrothecols G and H (**1** and **2**) were identified by the detailed analysis of relevant NOE correlations and the coupling constants, combining with the theoretical conformational analysis. The absolute configuration of penicilliumin A was suggested to be the same as that of myrothecol H by the comparison with their ^13^C NMR data of C-11 and C-6′.

## Supplementary Files

Supplementary File 1
