# Supplementary material for: Myrothecols G and H, Two New Analogues of the Marine-Derived Quinone Sesquiterpene Penicilliumin A
Source: Mar Drugs. 2015 May 27;13(6):3360–7. doi: 10.3390/md13063360 (PMC4483633; doi:10.3390/md13063360)
Supplement: Supplementary File 1 [file marinedrugs-13-03360-s001.pdf]

Supplementary Information

Table S1. Relative and free energies[a] and equilibrium populations[b] of low-energy conformers of **1** and **2** in MeOH.

Figure S1. Low-energy conformers of 1′*S*-isomer. Fragment from C-1 to C-4 was removed and replaced by hydrogen atoms in order to save computation time.

Figure S2. Low-energy conformers of 1′*R*-isomers. Fragment from C-1 to C-4 was removed and replaced by hydrogen atoms in order to save computation time.

Figure S3. ^{1}H NMR spectrum of Myrothecol G (**1**)

Figure S4. ^{13}C NMR spectrum of Myrothecol G (**1**)

Figure S5. HSQC spectrum of Myrothecol G (**1**)

Figure S6. ^{1}H-^{1}H COSY spectrum of Myrothecol G (**1**)

Figure S7. HMBC spectrum of Myrothecol G (**1**)

Figure S8. NOESY spectrum of Myrothecol G (**1**)

Figure S9. HRESIMS spectrum of Myrothecol G (**1**)

Figure S10. ^{1}H NMR spectrum of Myrothecol H (**2**)

Figure S11. ^{13}C NMR spectrum of Myrothecol H (**2**)

Figure S12. HSQC spectrum of Myrothecol H (**2**)

Figure S13. ^{1}H-^{1}H COSY spectrum of Myrothecol H (**2**)

Figure S14. HMBC spectrum of Myrothecol H (**2**)

Figure S15. NOESY spectrum of Myrothecol H (**2**)

Figure S16. HRESIMS spectrum of Myrothecol H (**2**)

Table S1. Relative and free energies [a] and equilibrium populations [b] of low-energy conformers of **1** and **2** in MeOH.

Conformer	Δ*E*	Δ*G*	*P* (%)
1′*S*-isomers			
1a1	0.56	0.0	42.8
1a2	0.60	1.17	6.0
1a3	0.77	1.44	3.8
1b1	0.0	0.15	33.0
1b2	0.26	1.06	7.1
1b3	0.35	1.30	4.7
1c	0.92	1.65	2.6
1′*R*-isomers			
2a1	0.00	0.00	75.6
2a2	0.98	0.65	6.2
2a3	1.19	1.24	5.0
2b1	1.76	1.76	8.3
2b2	2.47	1.96	2.5
2b3	2.75	2.54	2.3

[a] At the B3LYP/def2-TZVP level, in kcal/mol; [b] From Δ*G* values at 298.15 K.

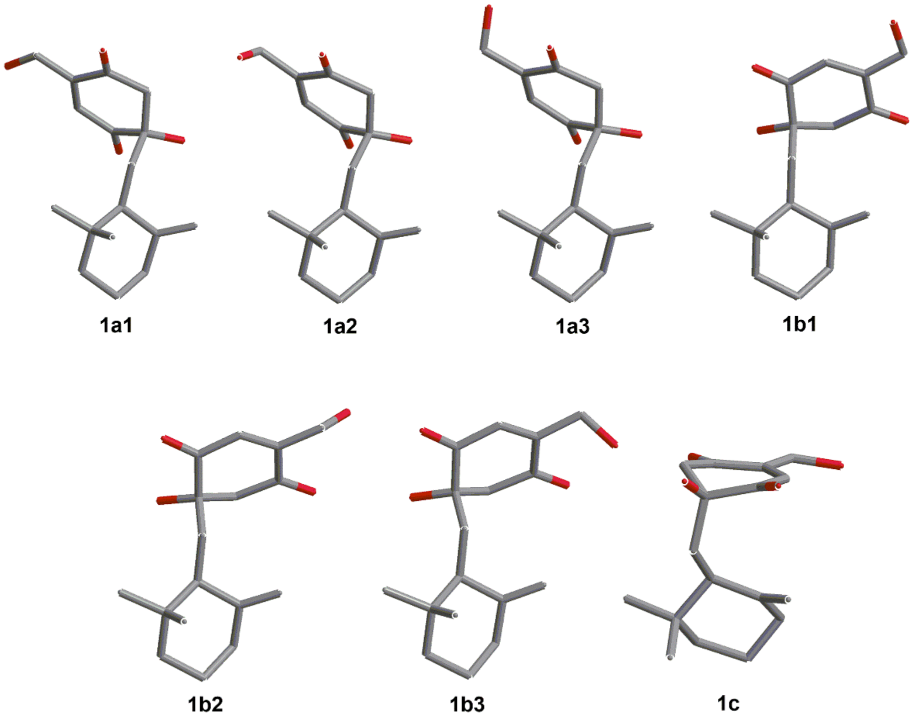

Figure S1. Low-energy conformers of 1′*S*-isomers. Fragment from C-1 to C-4 was removed and replaced by hydrogen atoms in order to save computation time.

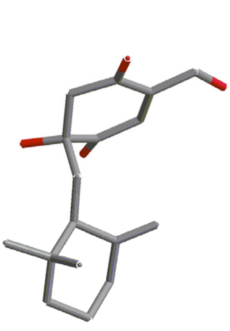

2a1

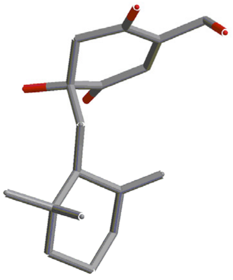

2a2

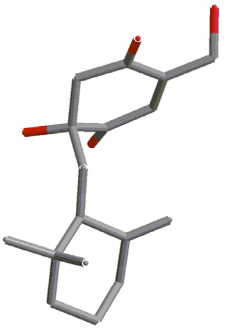

2a3

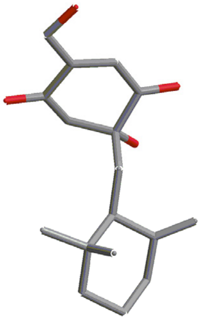

2b1

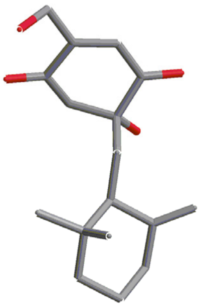

2b2

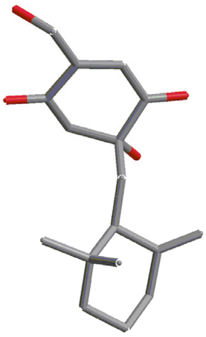

2b3

Figure S2. Low-energy conformers of 1′*R*-isomers. Fragment from C-1 to C-4 was removed and replaced by hydrogen atoms in order to save computation time.

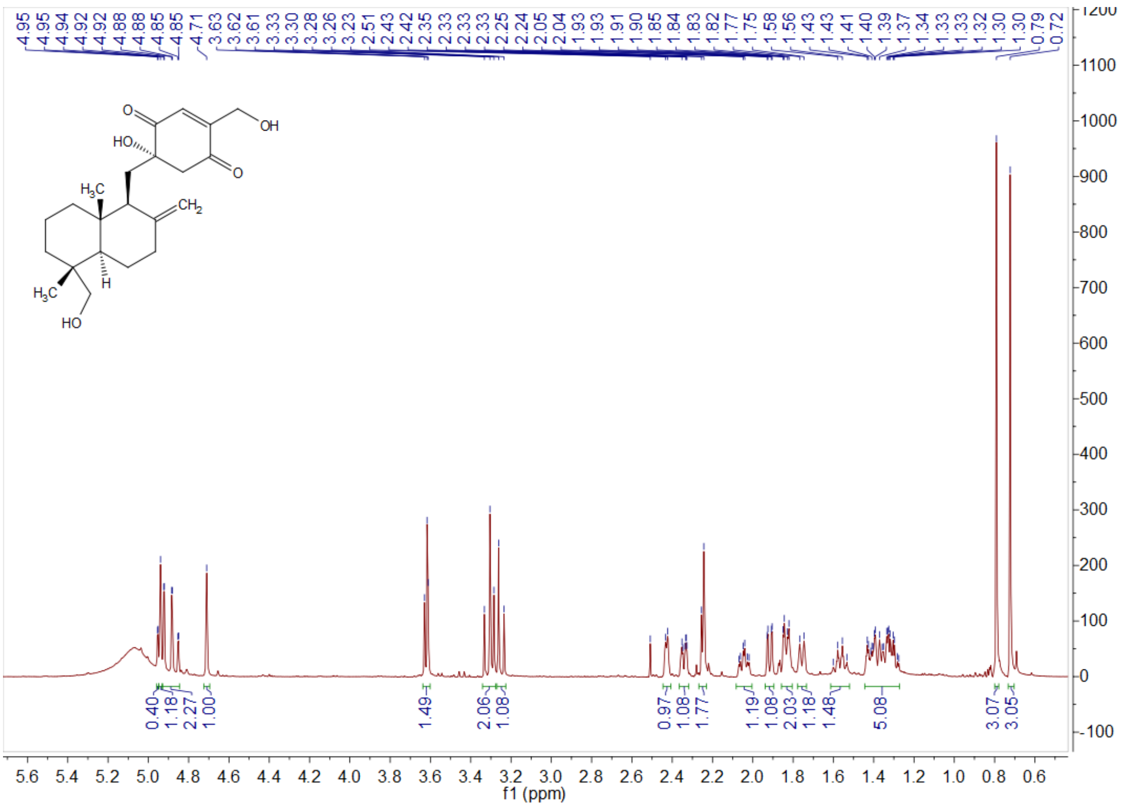

Figure S3. ^{1}H NMR spectrum of Myrothecol G (**1**).

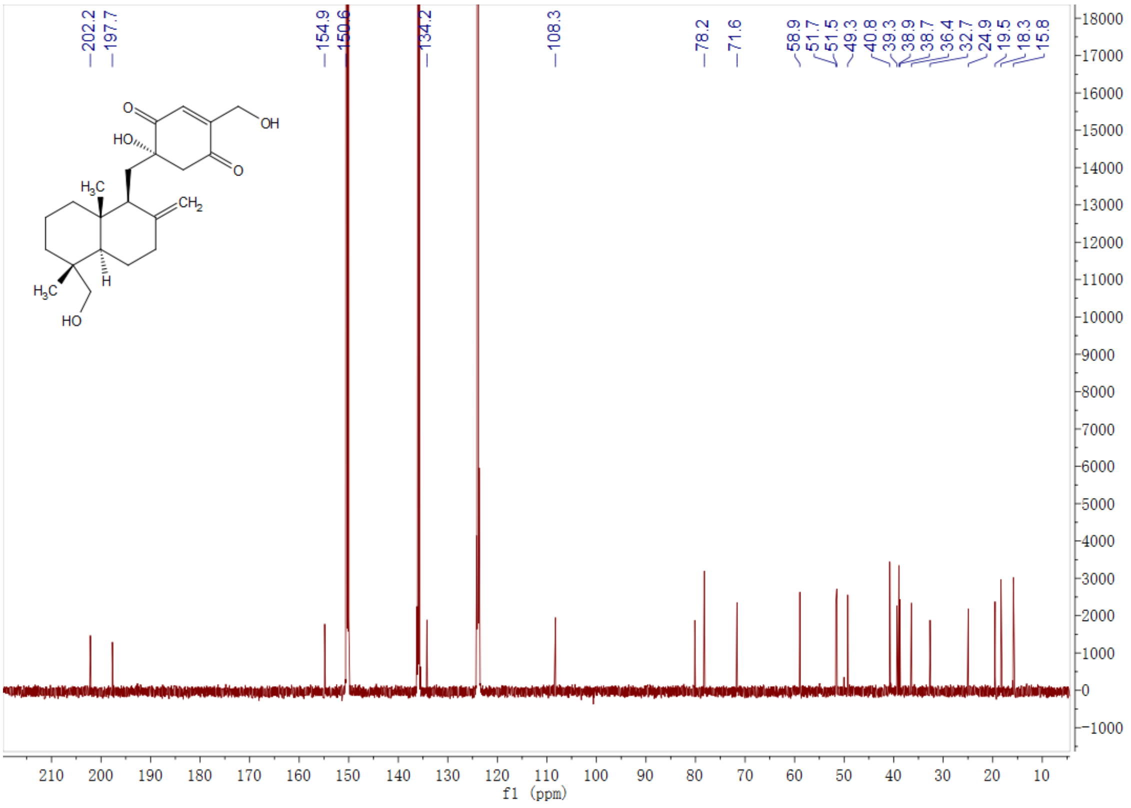

Figure S4. ^{13}C NMR spectrum of Myrothecol G (**1**).

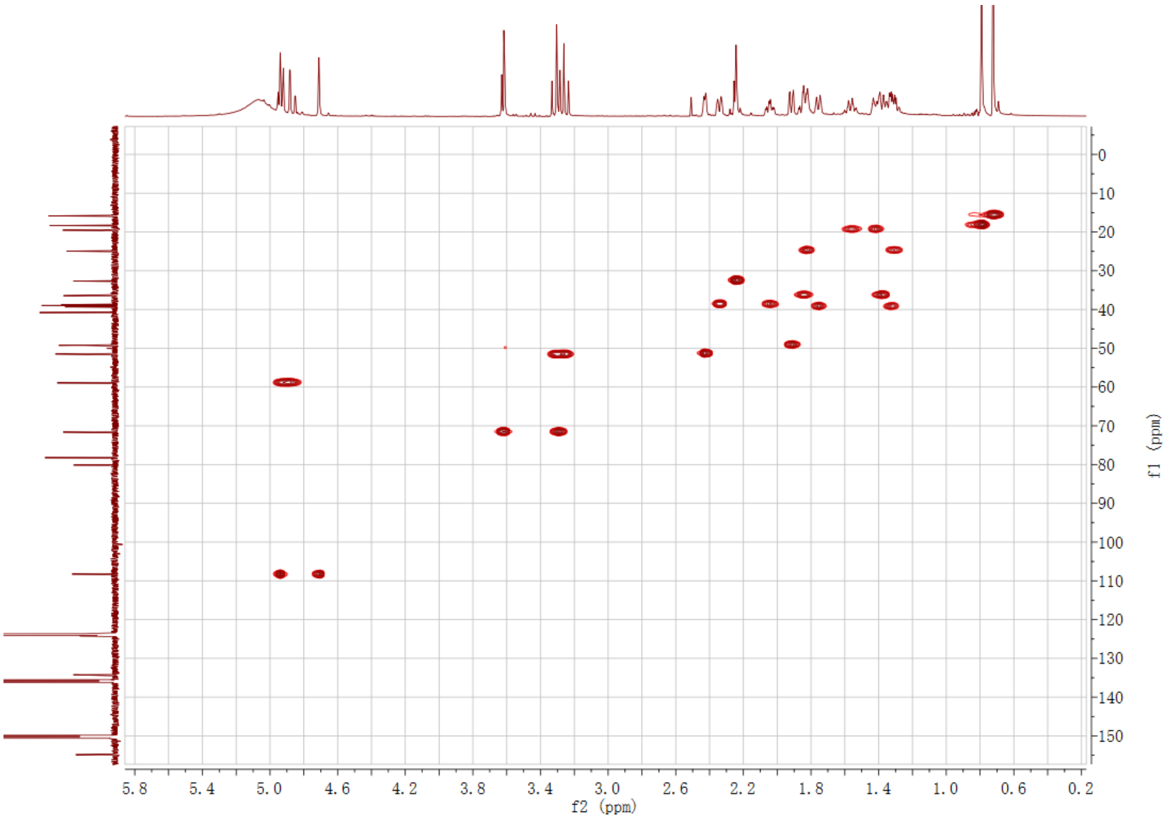

Figure S5. HSQC spectrum of Myrothecol G (**1**).

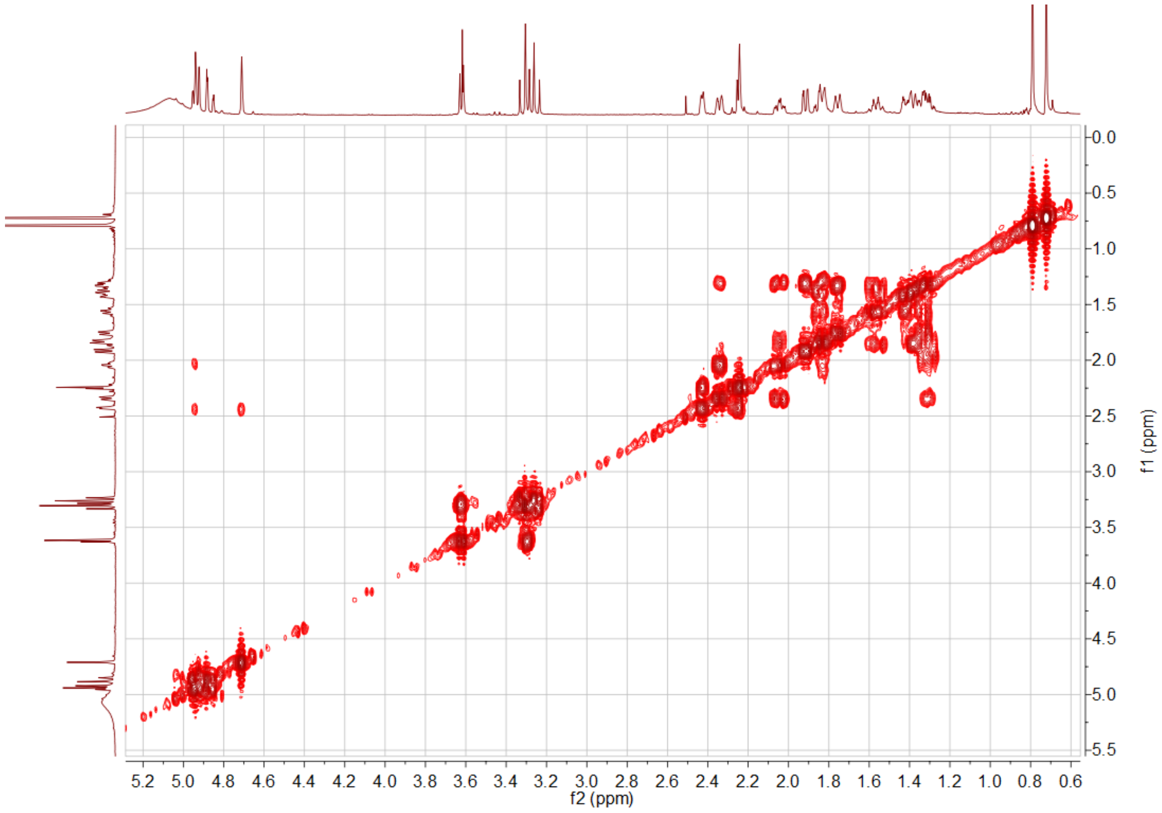

Figure S6. ^{1}H–^{1}H COSY spectrum of Myrothecol G (**1**).

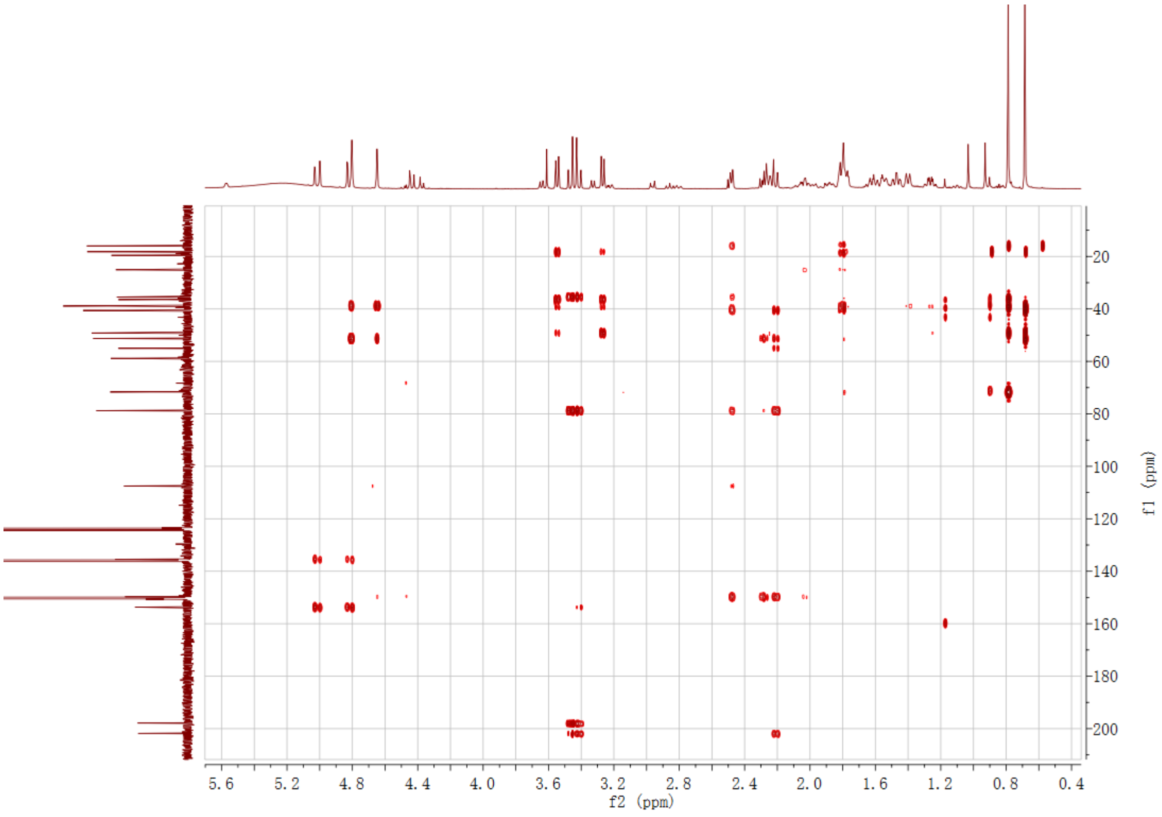

Figure S7. HMBC spectrum of Myrothecol G (**1**).

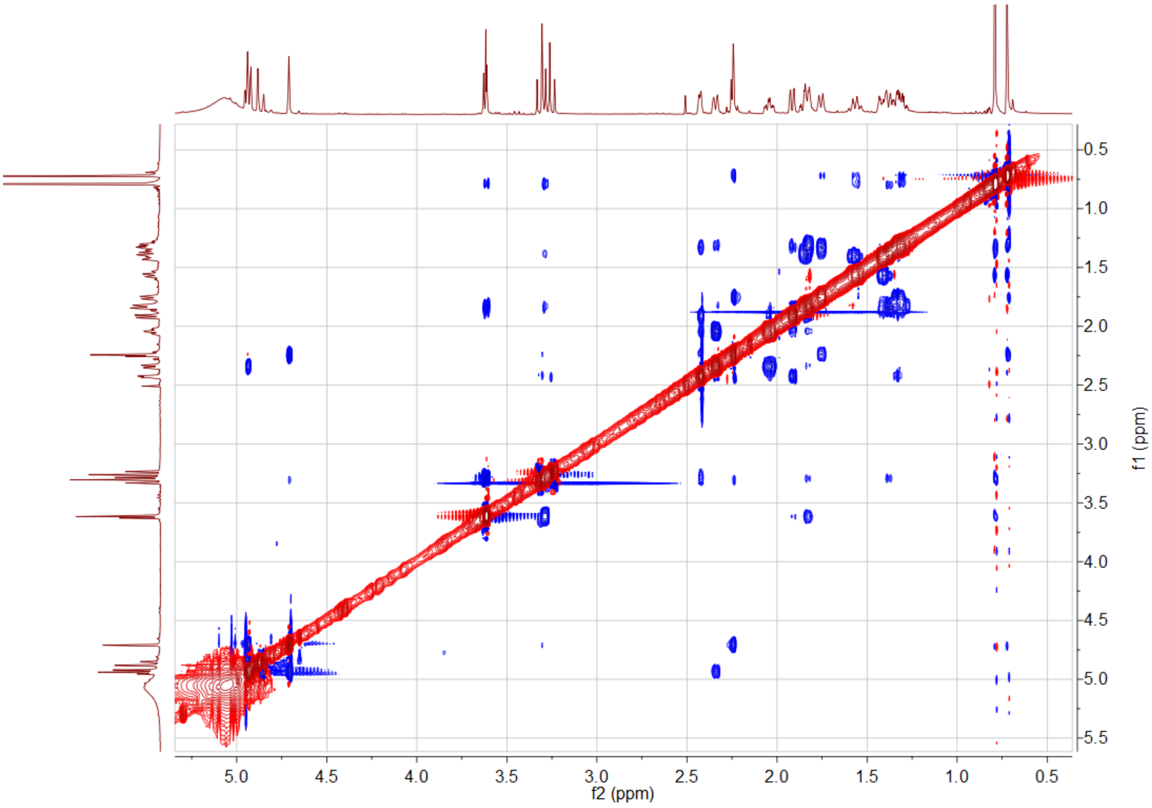

Figure S8. NOESY spectrum of Myrothecol G (**1**)

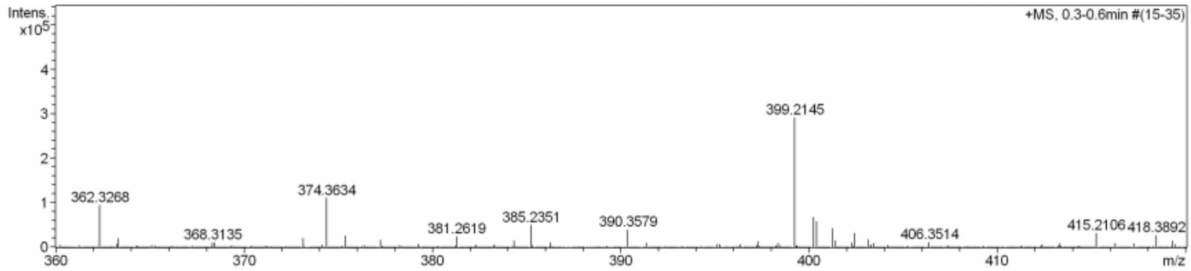

Meas. m/z	#	Formula	Score	m/z	err [mDa]	err [ppm]	mSigma	rdb	e^- Conf	N-Rule
377.2319	1	C 22 H 33 O 5	100.00	377.2323	0.4	1.0	240.2	6.5	even	ok
399.2145	1	C 22 H 32 Na O 5	100.00	399.2142	-0.3	-0.7	60.7	6.5	even	ok

Figure S9. HRESIMS spectrum of Myrothecol G (**1**).

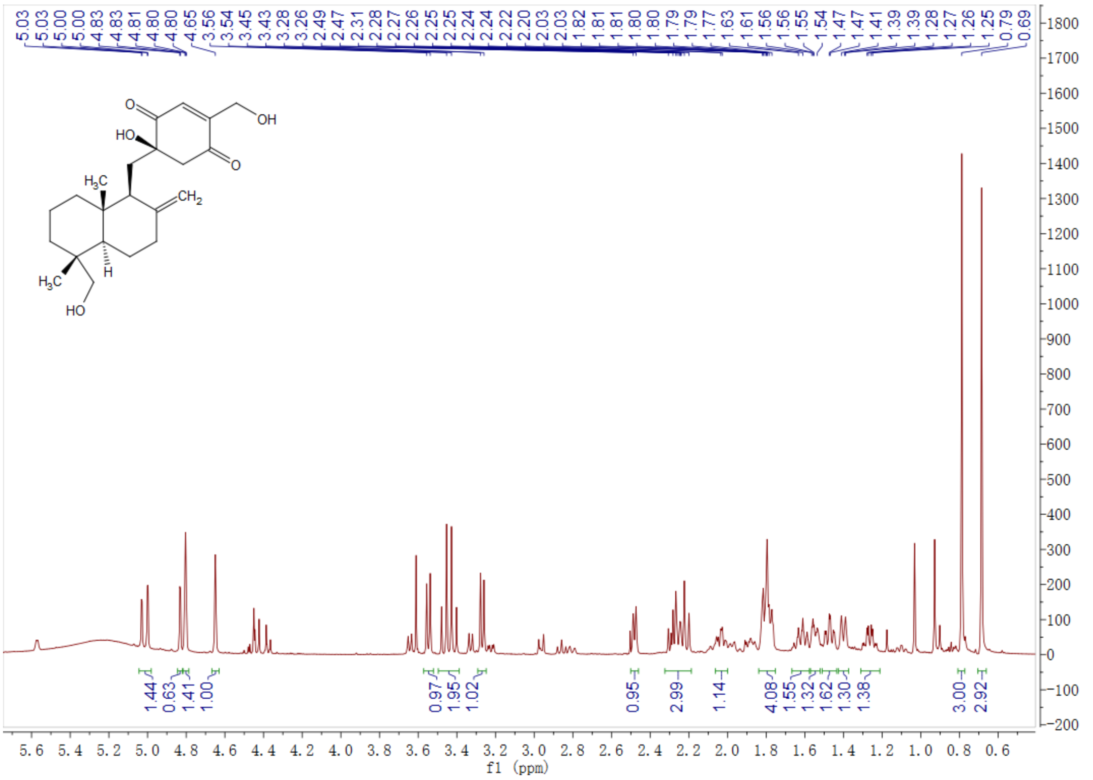

Figure S10. ^{1}H NMR spectrum of Myrothecol H (**2**).

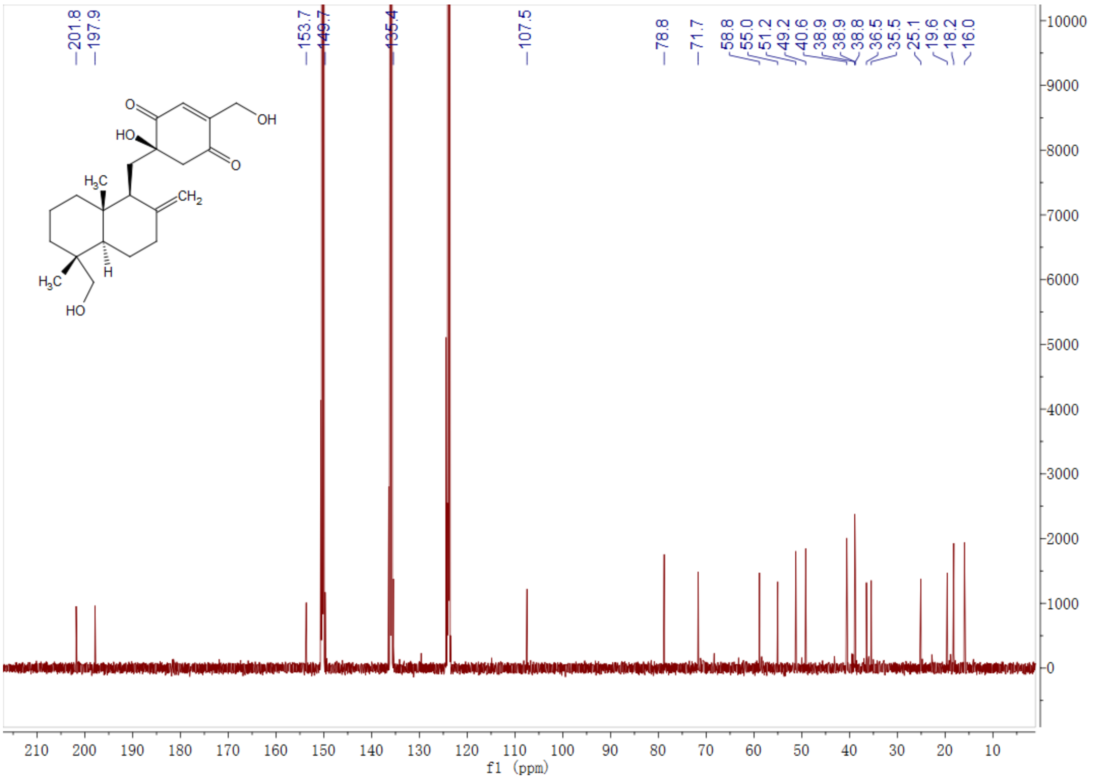

Figure S11. ^{13}C NMR spectrum of Myrothecol H (**2**).

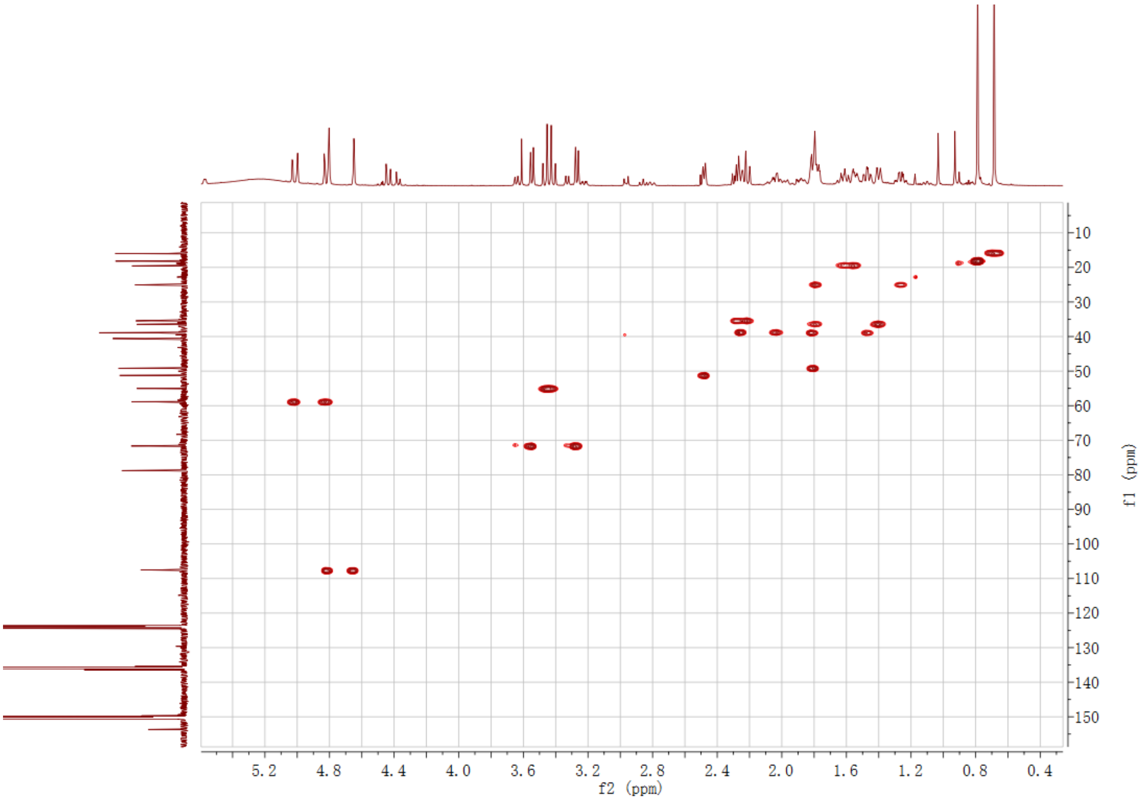

Figure S12. HSQC spectrum of Myrothecol H (**2**).

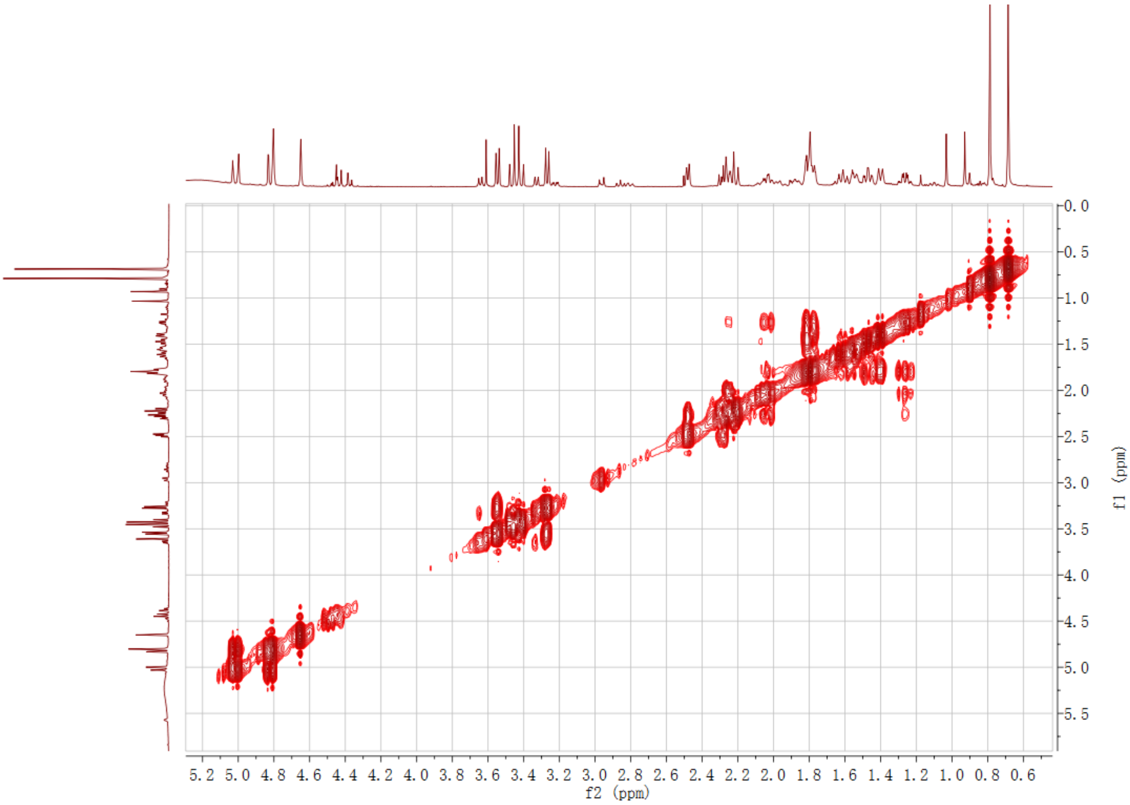

Figure S13. ^{1}H–^{1}H COSY spectrum of Myrothecol H (**2**).

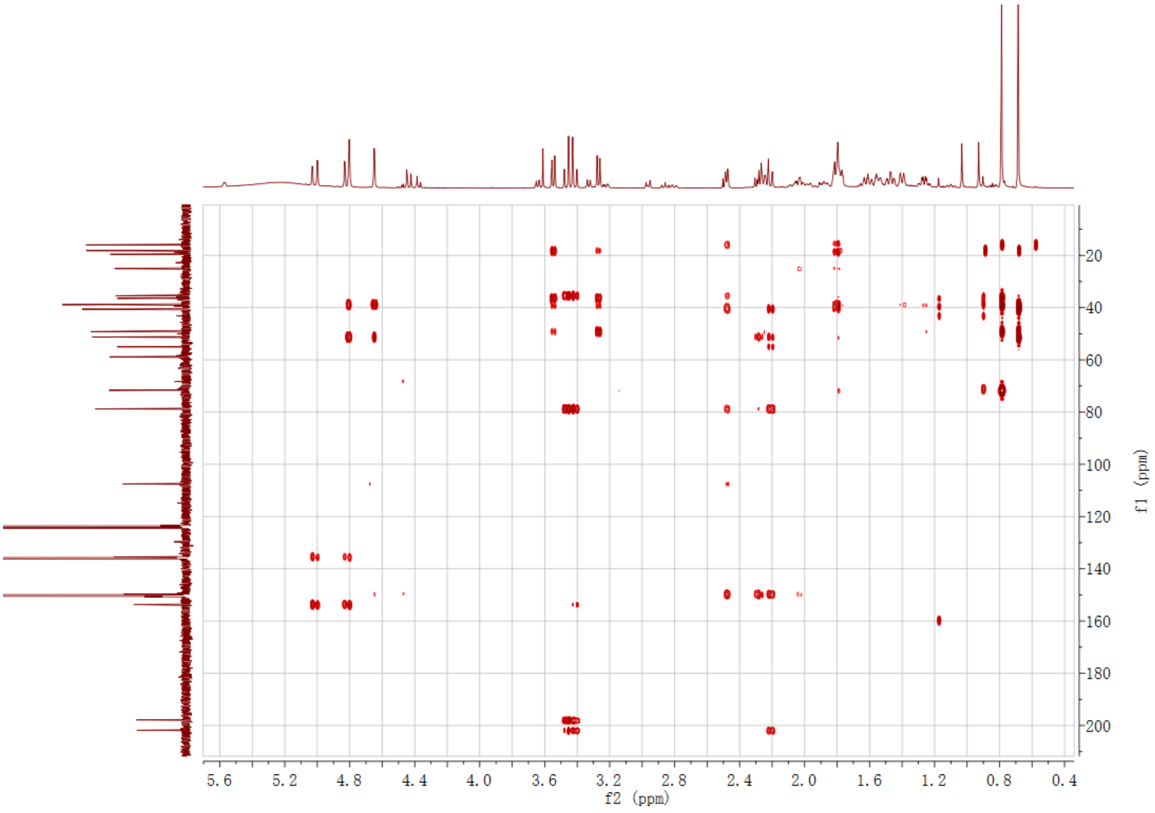

Figure S14. HMBC spectrum of Myrothecol H (**2**).

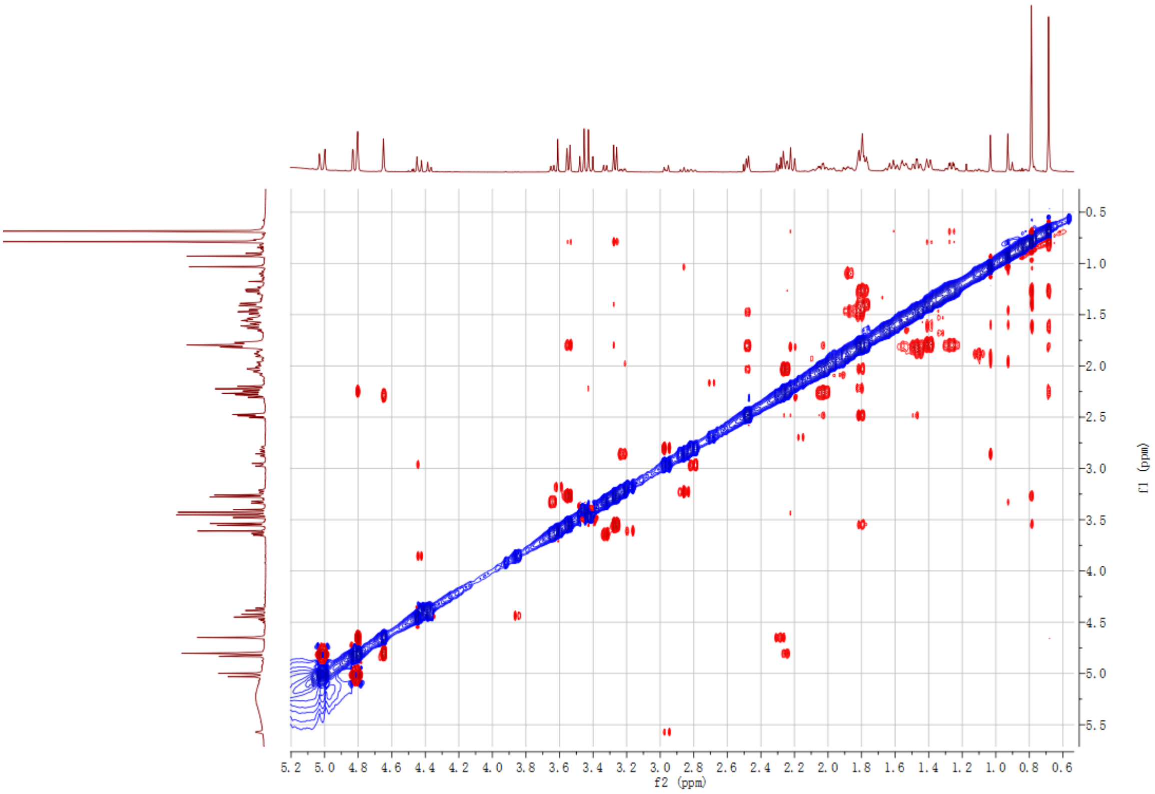

Figure S15. NOESY spectrum of Myrothecol H (**2**).

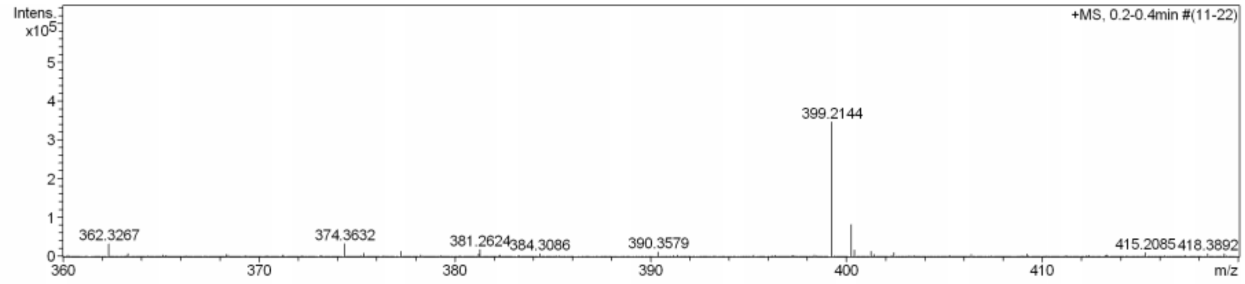

Meas. m/z	#	Formula	Score	m/z	err [mDa]	err [ppm]	mSigma	rdb	e⁻ Conf	N-Rule
377.2322	1	C 22 H 33 O 5	100.00	377.2323	0.1	0.3	81.5	6.5	even	ok
399.2144	1	C 22 H 32 Na O 5	100.00	399.2142	-0.2	-0.5	3.0	6.5	even	ok

Figure S16. HRESIMS spectrum of Myrothecol H (**2**).

© 2015 by the authors; license MDPI, Basel, Switzerland. This article is an open access article distributed under the terms and conditions of the Creative Commons Attribution license (http://creativecommons.org/licenses/by/4.0/).
